# Ovarian hormones and eating disorders

**DOI:** 10.3389/fpsyg.2024.1467795

**Published:** 2024-09-09

**Authors:** John K. Young

**Affiliations:** Department of Anatomy, Howard University College of Medicine, Washington, DC, United States

**Keywords:** anorexia nervosa, bulimia, estrogen, progesterone, polycystic ovaries, treatment

## Abstract

The eating disorders anorexia nervosa and bulimia nervosa are much more common in women than in men. Also, there is evidence for a role of gene mutations in these disorders. This review examines recent data about the possibility that ovarian estrogens may contribute to the symptoms of anorexia nervosa and partly account for the sex difference in incidence of this disorder. Possible mechanisms linking genes that are abnormal in anorexia to pathways that could produce abnormal responses to estrogen are also examined. In addition, recent data pointing to a role of ovarian androgens in the symptoms of bulimia nervosa are reviewed. These data may point to more effective adjustments for the therapy of these difficult to treat disorders.

## Introduction

This aim of this review is to examine recent information about the possible relationship between ovarian hormones and two eating disorders, anorexia nervosa and bulimia nervosa.

Anorexia nervosa is characterized by a persistent restriction in food intake, leading to dangerously low body weight. Despite decades of research, a consensus about the etiology and successful treatment of anorexia nervosa remains an elusive goal. A recent review by Guttierez and Carrera (2021) demonstrates the inefficacy of most treatment regimens for this disorder. A better understanding of the etiology of anorexia would therefore be of service.

Two features of anorexia are particularly challenging to understand. The first feature is a remarkable sex difference in incidence, with females predominating over males by at least nine to one. Moreover, the onset of anorexia often occurs around the time of puberty. The mean age of onset has been found to be about 15.9 years, with only a tiny fraction of 9,335 reviewed cases occurring earlier than 10 years of age (Watson et al., 2021—see Supplementary Figure S1). Also, an early puberty increases the risk for developing anorexia (Klump, 2013). These data suggest that the psychosocial and genetic identity of a female patient may not be as important for the risk of developing anorexia as is the activation of ovarian function and secretion of ovarian hormones during puberty.

A second puzzling feature of anorexia is the ability of patients to achieve extremely low body weights that they maintain over years. Millions of dieters strive to lose body weight each year, but few are able to achieve this goal (Grodstein, 1996). What special features of anorexia allow the maintenance of such low body weights?

An involvement of sex hormones in this disorder may help explain these two puzzling features. Estrogens have depressive effects upon body weight and food intake in both experimental animals and in humans (Hirschberg, 2012; Santollo and Daniels, 2015). Thus, it has been proposed that an abnormal response to rising levels of estrogens during puberty may contribute to anorexia (Klump et al., 2010; Timko et al., 2019; Young, 1975; Young, 2010). Since these proposals were published, more data have recently become available that would help an analysis of these hypotheses.

### Anorexia nervosa

While the focus of this review is upon biological factors in anorexia nervosa, it should from the outset be acknowledged that psychological factors doubtless contribute to the etiology of anorexia. For example, during the 1980s, a researcher named Hilde Bruch proposed that patients with anorexia experience a distorted body image. This proposal, while dated, still commands widespread acceptance in both the clinical community and in the general population; a recent review demonstrates the marked influence of these proposals upon current views of anorexia (Treasure and Cardi, 2017). This viewpoint is in conflict with more biological explanations of the disorder reviewed in this paper. However, a recent review noted that, prior to numerous lectures and papers by Dr. Bruch, a distorted body image was never reported in the scientific literature on anorexia. Instead, features such as abdominal discomfort were prominent (Guttierez and Carrera, 2021). Thus, it may be premature to dismiss biological explanations for anorexia in favor of purely psychological ones.

Depression and irritability, along with episodes of hyperactivity, are often found in anorexia. However, many researchers, including Hilde Bruch, have felt that some of these traits may be secondary to starvation and loss of body fat (Treasure and Cardi, 2017). In support of this, administration of a fat cell hormone, leptin, to anorexic patients appears to affect brain function and has a positive effect upon these symptoms (Hebebrand et al., 2022).

Genetics plays a major role in anorexia. Early studies demonstrated that identical twin girls were frequently concordant for anorexia, but fraternal twins rarely developed that disorder simultaneously. This lead to extensive searches for abnormalities in candidate genes that could contribute to anorexia. Initially, a disturbed function of dopaminergic or serotoninergic circuits were hypothesized to underlie anorexia. However, many of these genetic studies were not confirmed with larger sample sizes of patients. The current view is that many genes, probably differing between individuals, may somehow lead to the same endpoint or phenotype in anorexia, making an understanding of anorexia more complicated. Thorough reviews of all of the genes scrutinized in anorexia nervosa have been published recently (Baker et al., 2018; Donato et al., 2022).

In spite of these limitations, a number of genetic abnormalities recently found in anorexia may shed light upon the etiology of the disorder. One gene codes for a protein named the Estrogen Related Receptor alpha (ERRα). This protein does not bind estrogen, but does have the ability to interact with true estrogen receptor proteins. In some tissues, some ERR proteins can interfere with the ability of estrogen receptors to bind to DNA, effectively diminishing the cellular response to estrogen. A mutation in an ERR, causing it to become inactive, might therefore promote a hyperresponsiveness to estrogen (Cui et al., 2013; Saito and Cui, 2018; Tanida et al., 2015).

It might be objected that ERR proteins are not abundant in the arcuate nucleus of the hypothalamus, where estrogens influence feeding behavior. Thus, no interaction between ERR and estrogen receptors would be expected at this site. However, ERR proteins are abundant in the dorsal medulla and raphe nuclei, where estrogen also exerts effects upon food intake (Cui et al., 2013; Santollo and Daniels, 2015). So, an interaction between ERR proteins and estrogen receptors may in fact be applicable to anorexia nervosa.

Another gene implicated in anorexia nervosa codes for a protein called soluble epoxide hydrolase (sEH) (Shih et al., 2016). This protein is found in astrocytes throughout the brain and in neurons in the central amygdala (Marovsky et al., 2009). This enzyme converts epoxy-linked fatty acids into their corresponding diol forms. At first glance, it is difficult to understand why such a widely distributed enzyme might have effects specifically upon the control of feeding behavior. However, if one makes an assumption that abnormalities in this enzyme would have most prominent effects upon cells with unusually high rates of fatty acid metabolism, then it might be possible to construct a mechanism which explains an involvement of sEH in anorexia.

Astrocytes with high rates of fatty acid metabolism possess a protein called Fatty Acid Binding Protein 7 (FABP7). An abnormality in sEH might have particularly strong effects upon the function of FABP7+ cells. These cells are very abundant in the arcuate nucleus of the hypothalamus but are rare in other brain regions. FABP7+ astrocytes are in close contact with leptin-sensitive arcuate neurons that regulate feeding behavior; these astrocytes modulate the function of leptin-sensitive neurons (Yamamoto, 2018; Young, 2002). Thus, an abnormality in sEH might be expected to have unusually potent effects upon FABP7+ astrocytes and upon lipid metabolism in hypothalamic circuits that regulate feeding.

Besides an abnormality in sEH, other manipulations can cause changes in hypothalamic lipid metabolism. One such manipulation is the feeding of a high fat diet. In rats, high fat diets induce changes in the function of leptin-sensitive neurons; these changes are influenced by glial cells (Guillebaud et al., 2020).

In this context, it is noteworthy that rats fed a high fat diet show a marked hyperresponsiveness to anorexic effects of estradiol (Young et al., 1979).

At least part of estrogen’s anorexic effects are mediated through leptin-sensitive neurons in the arcuate nucleus (Gonzalez-Garcia et al., 2023). Alterations in hypothalamic lipid metabolism, due either to mutations of sEH in humans or to exposure to a high fat diet in rats, could result in changes in the function of leptin-sensitive neurons and an abnormal response to anorexic effects of estrogen. Effects of high-fat diet feeding on the hypothalamus involves increases in proteins like FAPB7 and another protein named ERB, which has also found to be abnormal in anorexia nervosa (Adlanmerini, 2021; Baker et al., 2018).

While the exact mechanism for the enhanced anorexic effects of estrogens in fat-fed rats has not been determined, it may involve the ability of a high-fat diet to reduce the reactivity of hypothalamic neurons to leptin. Since estrogen can increase hypothalamic sensitivity to leptin, perhaps the restoration of leptin sensitivity to baseline by estrogen might have more dramatic effects in fat-fed rats than in control rats (Gonzalez-Garcia et al., 2023).

Any validation of this speculation about genes related to anorexia must require experimental investigation. For example, examination of anorexic effects of estrogen in experimental animals genetically modified to express abnormalities in ERR or in sEH would be one means of exploring this question. These genetic pathways by no means represent the only mechanisms that could alter responsiveness to estrogen. For example, exposure of the brain to steroids during early development can lead to long-lasting alterations in responsiveness to estrogen and could represent another risk factor for anorexia (Barakat et al., 2023). Many other contributing factors can be proposed (Young, 2010).

Is there any concrete evidence that estrogen could provoke an abnormal regulation of feeding in women? In fact, such evidence can be derived from patients with a chromosomal abnormality called Turner’s syndrome. In this disorder, ovaries fail to develop during embryogenesis. As a consequence, patients do not undergo a normal puberty. A common approach to this problem is to treat patients with ethinyl estradiol to produce secondary signs of adolescence. In about 20 cases reported in the literature, estradiol treatment evokes symptoms of weight loss and anorexia that are indistinguishable from spontaneous cases of anorexia nervosa (reviewed in Muhs and Liberz, 1993; Young, 2010). Symptoms disappear upon cessation of treatment with estradiol.

Why would selected Turner’s patients react to estradiol in this way? Abnormalities in multiple organ systems are found in this syndrome, so it is difficult to pinpoint any precise reason for this response to estrogen.

Turner’s patients are known to sometimes show depressed levels of circulating thyroxine, due to an autoimmune attack upon the thyroid gland (Witkowska-Sędek et al., 2017). Since thyroxine depresses the response to anorexic effects of estradiol, a low level of thyroxine could conceivably lead to an elevated response to estradiol (Young, 1986). This modulation of anorexic effects of estrogen by thyroxine most likely involves an interaction between estrogen receptors and thyroxine receptors in the hypothalamus (Holland et al., 1998).

All of this information suggests a role for ovarian estrogens in the etiology of anorexia nervosa. One objection to this proposal is that blood levels of estrogen tend to be low in anorexic patients, since their depressed body weight can be associated with amenorrhea. About 60–80% of patients with anorexia have starvation-induced amenorrhea and lowered blood levels of estrogens. Other endocrine changes (pituitary–adrenal axis, pituitary-thyroid axis, bone metabolism, etc.) have also been noted (Haines, 2023). These endocrine changes complicate the analysis of the etiology of anorexia.

In spite of a lowered secretion of estrogen, it is nevertheless possible that even these lowered levels could have a behavioral effect. For example, it is unclear if the anorexic properties of estrogens show a linear relationship with blood levels of estrogens. In rats, administration of low doses of estrogen over several days has an effect upon food intake that is similar to administration of a single, larger dose (Young et al., 1979). Also, in rats, even very low, prepubertal blood levels of estrogen still have the ability to influence food intake (reviewed in Young, 2010). Thus, even lowered levels of estrogen seen in females with amenorrhea or even male anorexic patients could nevertheless exert some biological effects.

There is evidence that ovarian hormones can produce changes in mood, negative emotions, and subjective feelings of malaise over the course of the ovarian cycle (Finch et al., 2023; Gustavson et al., 1989; Payne, 2003). If patients suffering from anorexia also experience an intensification of these reactions to sex hormones, then the blunting of hormone secretion produced by self-starvation could provide a welcome relief from these subjective feelings. This might provide one additional explanation for why a state of self-starvation, so aversive to most people, could become unconsciously self-reinforcing in anorexia and allow the maintenance of extremely low body weights.

This line of reasoning is of theoretical interest, but is of little practical value unless it leads to some sort of treatment that could reduce the suffering and high mortality rate found in anorexic patients. If estrogen does indeed contribute to the symptoms of anorexia nervosa, then one type of treatment might be helpful. Administration of progestins blocks anorexic effects of estrogen and so could represent one pharmacological means of therapy (Gray and Wade, 1981).

Even if the hypothesis presented in this review proves to be invalid, progestins still could represent a possible means of therapy. Administration of some types of progestins has proven to be an effective and relatively benign means of increasing body weight and appetite in patients suffering from AIDS or cancer (Argiles et al., 2013; Currow et al., 2021; Mateen and Jatoi, 2006; Von Roenn et al., 1994). Some caution should be utilized in the administration of progestins, since a long-term use of these hormones may have adverse effects upon bone density (Busen, 2004). Perhaps a brief exposure to progestins could interrupt the self-reinforcing cycle of weight loss and improve outcome, while avoiding side effects of progestins.

Some patients with anorexia doubtless are prescribed contraceptive hormones at some points in their lives. However, since most contraceptives are of the combined estrogen-progesterone type, effects of progestagen-only medications upon the course of anorexia may not yet have been completely examined.

### Bulimia nervosa and binge eating disorder

According to the Diagnostic and Statistical Manual of Mental Disorders, binge eating disorder can be defined as a propensity for poorly controlled episodes of overeating that can result in weight gain. In bulimia nervosa, episodes of overeating are accompanied by self-induced vomiting or use of laxatives to reduce the weight gain (Donato et al., 2022). The overall prevalence of binge eating disorder and bulimia within the general population seems to be somewhat higher than that for anorexia; the sex difference in incidence, while still favoring females, seems to be less than that for anorexia.

A strong influence of genetics has also been identified for bulimia. Thus far, no definitive candidate genes have been identified, although genes for appetite regulating circuits utilizing neurotransmitters like serotonin, dopamine, endocannabinoids, etc., have been examined (Donato et al., 2022).

Recent studies have found that phases of the menstrual cycle appear to influence episodes of binge eating (Klump et al., 2014; Finch et al., 2023; Rolan et al., 2023).

Perhaps the most interesting recent report is a study of women suffering from polycystic ovary syndrome (PCOS). This syndrome results from an abnormal pattern of hormone release from the pituitary gland, which causes the enlargement of ovarian follicles but fails to stimulate ovulation and the expulsion of oocytes from the ovaries. Enlarged follicles form cysts that continue to produce steroid hormones. Women with PCOS have an increased risk for developing eating disorders. In particular, the rates of bulimia and binge eating disorder were markedly elevated in women with PCOS compared with controls. A possible cause for this association may be an overproduction of ovarian androgens, which have a stimulatory effect upon appetite and body weight. Ovarian androgens also likely represent an explanation for symptoms of hirsutism and skin blemishes that are frequent features of PCOS (Lalonde-Bester et al., 2024). Since other factors may also contribute to bulimia in PCOS, androgens may not represent the sole explanation for these findings.

A common treatment for PCOS is the administration of combined estrogen-progestagen type contraceptives, which suppress gonadotropin release from the pituitary in the management of PCOS. This report suggests that further study of the role of androgens in bulimia would be warranted.

## Discussion

Substantial evidence has accumulated suggesting that ovarian estrogens make a contribution to the symptoms of anorexia. If so, a potential explanation for the marked sex difference in incidence of anorexia nervosa may be apparent. Medications that counteract effects of estrogens upon food intake and body weight should be evaluated as potential components of the treatment of anorexia.

Data from women with polycystic ovarian syndrome also indicate a role for ovarian hormones in the etiology of binge eating disorder and bulimia nervosa. Further study of the role of ovarian androgens in the etiology of bulimia would be desirable.
